# Evaluating an Integrated Local System Response to the COVID-19 Pandemic: Case Study of East Toronto Health Partners

**DOI:** 10.5334/ijic.7014

**Published:** 2023-06-22

**Authors:** Sara Shearkhani, Donna Plett, Jeff Powis, Catherine Yu, Janine McCready, Lucy Lau, Phillip Anthony, Kate Mason, Kathleen Foley, Denny Petkovski, James Callahan, Laurie Bourne, Wolf Klassen, Anne Wojtak

**Affiliations:** 1 Michael Garron Hospital, CA; 2 East Toronto Health Partners, CA; 3 University of Toronto, CA; 4 St. Michael’s Hospital, CA; 5 South Riverdale Community Health Centre, CA

**Keywords:** integrated care, COVID-19, distributed leadership, community partnerships

## Abstract

**Introduction:**

East Toronto Health Partners (ETHP) is a network of organizations that serve residents of East Toronto, Ontario, Canada. ETHP is a newly formed integrated model of care in which hospital, primary care, community providers and patients/families work together to improve population health. We describe and evaluate the evolution of this emerging integrated care system as it responded to a global health crisis.

**Description:**

This paper begins by describing ETHP’s pandemic response mapping out over two years of data. To evaluate the response, semi-structured interviews were conducted with 30 decision makers, clinicians, staff, and volunteers who were part of the response. The interviews were thematically analyzed, and emergent themes mapped onto the nine pillars of integrated care.

**Discussion:**

The ETHP pandemic response evolved rapidly. Early siloed responses gave way to collaborative efforts and equity emerged as a central priority. New alliances formed, resources were shared, leaders emerged, and community members stepped forward to contribute. Interviewees identified positives as well as many opportunities for improvement post-pandemic.

**Conclusion:**

The pandemic was a catalyst for change in East Toronto that accelerated existing initiatives to achieve integrated care. The East Toronto experience may serve as a useful guide for other emerging integrated care systems.

## Introduction

COVID-19 rapidly evolved into an unprecedented worldwide public health crisis [1]. Its ramifications were experienced globally, resulting in health system shock, debilitating effects on the economy, society, and daily life, and disproportionately ill effects on vulnerable populations [2,3]. Despite concerted vaccination efforts and public health measures, new variants and outbreaks continued to arise [4].

In Canada, prompt action — progressive implementation of public health measures — was taken to combat the health crisis, including travel restrictions, border screening, and quarantine procedures [5]. In Ontario, the pandemic coincided with a new model for organizing and delivering integrated care. In 2019, Ontario Health Teams (OHTs) were created to achieve integrated care models with connected and coordinated teams across the health and social care continuum [6]. The impetus for this health system transformation was accelerated by the pandemic [3]. The East Toronto Health Partners (ETHP) was one of the first OHTs named by the Ontario Ministry of Health (MOH) in 2019, shortly before the pandemic impacted Toronto. ETHP leveraged the groundwork that had been laid to meet the unprecedented challenges of the COVID-19 pandemic.

Integrated care is a relatively new phenomenon, and its success has been difficult to clearly assess [7,8]. Even though randomized controlled trials may be considered the gold standard, evaluations that incorporate relevant contextual factors may be more suitable in this case due to the complexity of integrated care [9]. This study adds to the literature by providing an evaluation of an integrated care system that incorporates contextual factors while highlighting themes that are relevant to other integrated care systems.

This study asks: “What has been the impact of implementing a community-wide integrated response to COVID-19?” The paper is divided into two main sections. The first provides a summary of ETHP’s testing and vaccination efforts and highlights its novel initiatives and strategies from March 2020 to March 2022. The second is a qualitative evaluation of the collective response to the pandemic from the perspectives of ETHP’s decision makers, clinicians, staff, and community members. These findings are then tied into the nine pillars of integrated care developed by the International Foundation for Integrated Care (IFIC) [10]. The findings will inform next steps in strengthening health system resilience and the acceleration of integrated health and social care within our OHT and be useful for other emerging integrated care systems.

## East Toronto Health Partners

ETHP consists of a network of over 100 organizations spanning East Toronto [3,11]. The governance for this collaborative model [12] consists of six (increased to eight in early 2023) core (anchor) partners, as well as patient, caregiver and community advisors. ETHP’s anchor partners represent the continuum of care, including acute, primary, social services, home care, long-term care and mental health. In collaboration with the anchor partners, dozens of other health and social care partners work together to achieve shared goals in the community, including inclusion and equity [12]. ETHP’s main objective is to co-design, implement, and deliver an integrated model of care in which hospital, primary care, community providers and patients and families work together as one coordinated team. Michael Garron Hospital (MGH) is the only acute care hospital in East Toronto and was the primary infection prevention and control expertise guiding the East Toronto response to COVID-19.

**Figure 1:**
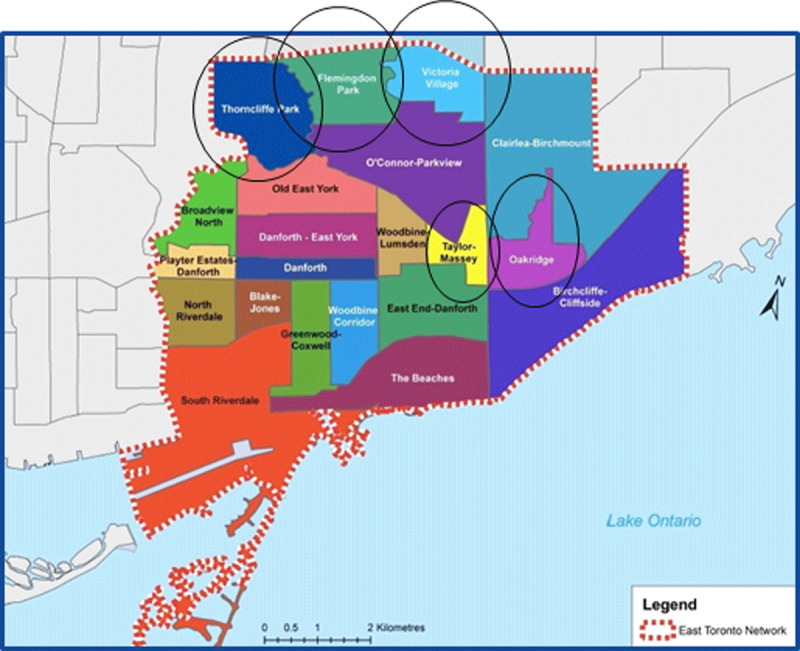
East Toronto’s 21 Neighbourhoods served by East Toronto Health Partners, including 5 Priority Neighbourhoods (circled).

East Toronto is home to approximately 300,000 residents living in 21 neighborhoods, including five priority neighbourhoods that have lower socioeconomic levels (Figure 1). Over 50 OHTs now exist across the province and ETHP is amongst the five OHTs with the most significant proportion of their residents living in less affluent neighborhoods [13]. Providing equitable access to care is a priority for ETHP and it has launched a neighbourhood model approach beginning with the five priority neighbourhoods.

### Description of ETHP’s COVID-19 response

**Figure 2:**
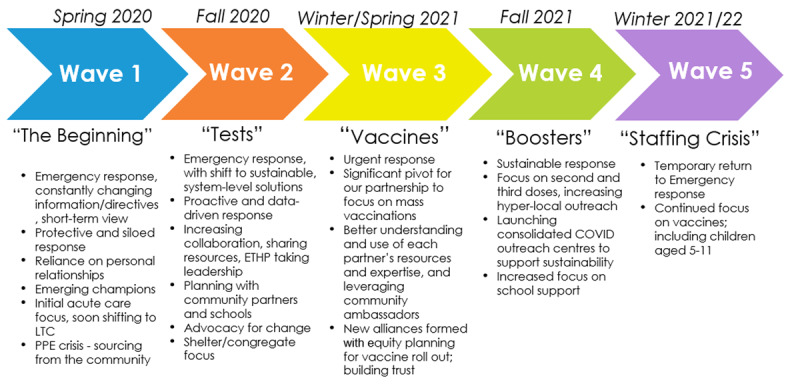
Main characteristics of the East Toronto Health Partners’ collective response to COVID-19.

In this section we summarize the characteristics of our response in each wave of the pandemic highlighting some of our novel initiatives and strategies (Figure 2).

#### Wave 1: The beginning

The early days of the pandemic were characterized by rapidly changing directives and a short-term, crisis mentality typical of emergency responses. Initially, organizations focused inwards on the needs of their staff and clients, resulting in a protective and siloed approach. Clinicians and primary care leaders were seen as the main source of knowledge, specifically on infection prevention and control (IPAC) and use of personal protective equipment (PPE). Personal relationships were particularly important in the early days to facilitate a collaborative response, however as the spread of the virus continued, new and innovative partnerships were required to enable a system-wide strategy. The first few weeks of the pandemic response focused on acute care, but this soon shifted to long term care (LTC) as outbreaks in these settings increased and care homes lacked the resources to prevent spread – 80% of COVID-19 deaths in Ontario were LTC residents in the first wave [14]. ETHP’s main strategy was to build internal capacity within LTC to enable them to make informed prevention and outbreak decisions. Additionally, the staffing crisis and the need to provide high-acuity care in LTC led to the development of a hospital-at-home philosophy where hospital staff were embedded within LTC homes. Addressing shortages of PPE for healthcare staff and community members also dominated efforts during this time and a community drive for PPE was launched (Community Sewing Face Mask Project [15]).

#### Wave 2: Tests

**Figure 3:**
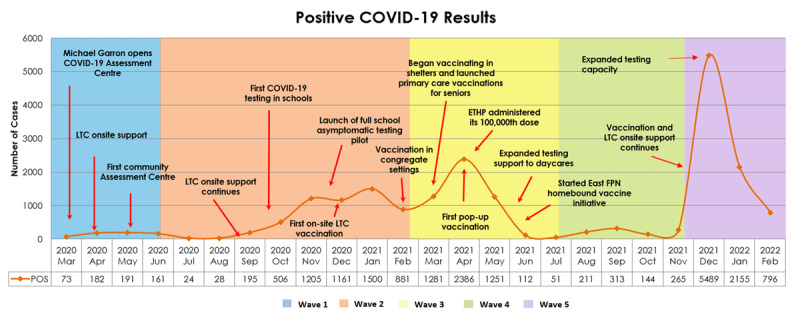
COVID-19 case counts in East Toronto from March 2020 to February 2022 and important milestones in the ETHP pandemic response.

By the second wave, the initial crisis response gave way to a more sustainable, systems-level, proactive, and data-driven response. Collaboration and resource sharing between organizations increased. The focal point of the response expanded beyond LTC to include communities and schools. Most initiatives during this time focused on testing and supporting self-isolation [16,17]. Figure 3 provides a timeline of key milestones in the ETHP response.

**Figure 4:**
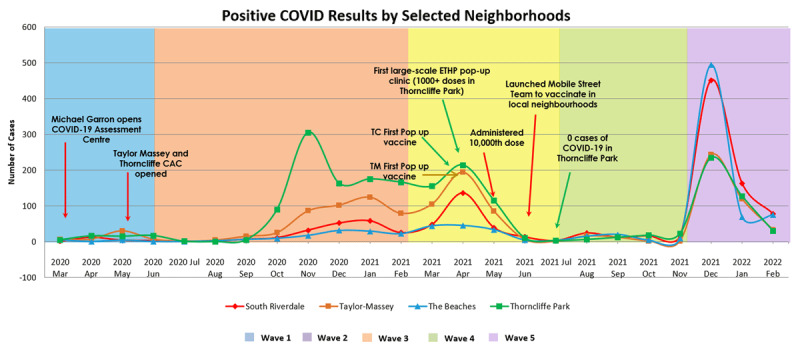
COVID-19 case counts in two priority neighbourhoods (Taylor-Massey and Thorncliffe Park) and two high SES neighbourhoods (The Beaches and South Riverdale) from March 2020 to February 2022 and important milestones in the ETHP pandemic response in priority neighbourhoods.

Providing equitable care established itself further as a key priority, especially as high case counts in disadvantaged neighborhoods led to disparate outcomes and stigmatization [18,19]. Two main approaches were used to bring an equity lens to the response. First, ETHP partners assertively advocated through local and national media for equitable distribution of resources and pushed provincial decision-makers to shift resources to hyper-local, equity-focused, and culturally appropriate efforts. The opening of one of Canada’s first community-based testing sites, which was combined with a food bank, was a direct result of community partnerships. Additionally, residents and local organizations launched “This is our story,” a social media campaign of news releases that highlighted the community’s resiliency, despite systemic barriers. Second, a neighbourhood model of care was employed in which individuals who tested positive or were exposed were provided with support, such as symptom management, referrals to other health, wellness, and social services, as well as financial and food supports needed for self-isolation. Figure 4 provides a timeline of key milestones of initiatives in priority neighbourhoods (e.g., Taylor-Massey and Thorncliffe Park), with two high socio-economic status neighbourhoods as a comparator (The Beaches and South Riverdale). While priority neighborhoods had the highest case counts early in the pandemic, after ETHP initiatives, case counts in these neighbourhoods were lower than in the two comparator neighbourhoods [20].

#### Wave 3: Vaccinations

**Figure 5:**
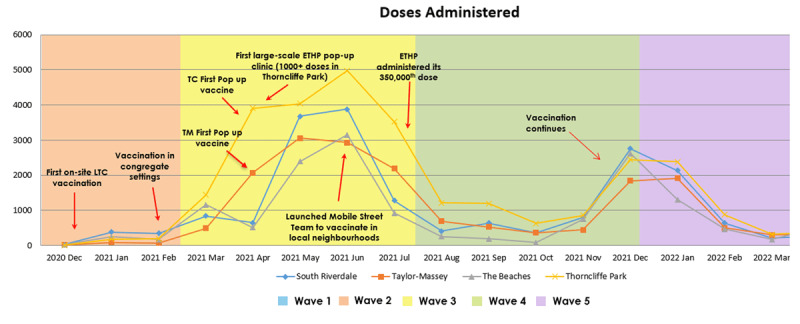
COVID-19 vaccination counts in two priority neighborhoods (Taylor-Massey and Thorncliffe Park) and two high SES neighborhoods (The Beaches and South Riverdale) from December 2020 to May 2022 and important milestones in the ETHP vaccination effort.

Vaccination efforts began when vaccines became available in December 2020. Phase 1 of the vaccine rollout in Toronto prioritized those aged 80+, seniors in congregate care settings, adults receiving home care, and health care workers [21]. In Phase 2, eligibility extended to neighbourhoods with high rates of infection, at-risk essential workers, and high-risk congregate living settings [22].The vaccination rollout marked a significant pivot for ETHP’s integrated ways of working. At this stage, it became clear that the wealth of knowledge residing within the community could help shape equitable delivery of vaccines, tailor outreach strategies, and combat vaccine hesitancy. A distributed leadership model was employed, which resulted in better understanding of community-based resources and expertise, which were then leveraged to advance collective goals. A particularly important and meaningful partnership was the collaboration with community members, some of whom volunteered as community ambassadors. This approach to leveraging community assets and resources is described in section 4.3.1. Community ambassadors played a key role in raising awareness and increasing vaccine confidence in residents, and improving vaccine inequity through ongoing rapid cycle change [23,24]. The vaccination effort also included vaccine clinics at schools to maximize accessibility. Overall, more that 612,308 doses have been administered in East Toronto. A timeline of key milestones related to the vaccination effort can be viewed in Figure 5.

#### Waves 4–6: Delta & Omicron

The fourth, fifth and sixth waves of the pandemic can each be characterized by their unique presentations. The severity of illness from Delta (wave 4) was mitigated through the timely administration of booster doses and increased support in the community (i.e., outreach centers, increased support for schools). Omicron (wave 5) reached new heights of transmission and coincided with significant staffing shortages as staff with mild illness were furloughed from the front line. Omicron subvariants were characterized by ongoing, propagated community transmission after the lifting of public health measures, alongside the integration of therapeutics into COVID-19 assessment clinics. Despite the distinct manifestations of each of these waves, a focus on sustainability and matured partnership models was consistent throughout. The initiatives and models of care developed through the pandemic became the norm. The relationships and partnership established early in the pandemic have matured and partners began to plan next steps, including building new partnerships and improving integration of social and healthcare models of care. The conversation changed from what ETHP can do for its organizations and clients to what and how it can contribute to all residents and advance an integrated care model that focuses on equity and the social determinants of health.

## Evaluating the ETHP Collective Response: Methods

The preceding section explicated the core initiatives of the ETHP COVID-19 response, which gives context for the evaluation. The multidisciplinary nature of a collective response is grounded in the ideologies, values, and beliefs of decision makers, clinicians, staff, and community members. As such, semi-structured interviews with 30 individuals were conducted to provide a richer understanding of the building blocks of the integrated COVID-19 response. Participants were decision makers, clinicians, staff, and community members who designed, implemented, led, and delivered ETHP’s COVID-19 strategies and initiatives since the pandemic began until the end of the fifth wave. They represented ETHP partners across the continuum of care. Purposive sampling was used to identify the participants [25]. The informants were identified in partnership with the ETHP’s governance committees. Snowball sampling was used when participants referred other key informants, and recruitment ended when saturation was reached (i.e., when no new themes were arising) [25].

**Figure 6:**
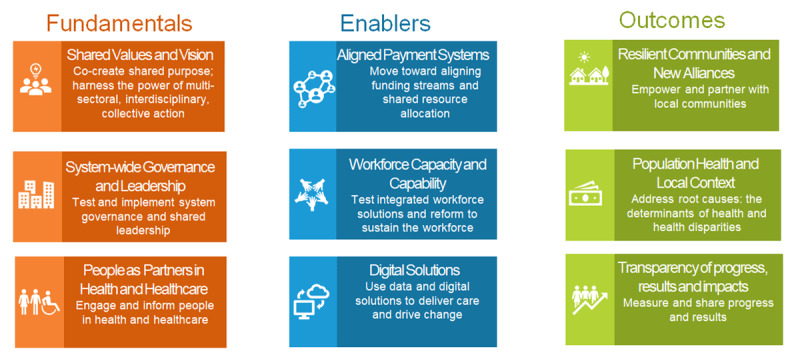
IFIC’s 9 pillars of integrated care.

One-on-one interviews were conducted over Zoom by two of the evaluation team members using a semi-structured interview guide (Appendix A). During the interview, participants reflected on how the collective response evolved over time; identified its main characteristics; reflected on what worked well and identified areas for improvement. Thematic analysis was used to analyse the interview data [25]. Analyses were conducted by the same evaluation team members who conducted the interviews. The team members began by reviewing and coding the transcripts independently. They met regularly and discussed emerging themes, similarities, and discrepancies. This process led to co-creation of a coding framework, which included main characteristics of ETHP’s response, areas for improvement, and implications for OHT development. The themes were mapped to the 9 pillars of integrated care developed by the International Foundation for Integrated Care [10] (Figure 6). Based on our findings, we further grouped the pillars to 3 overarching categories: fundamentals, enablers, and outcomes. Fundamentals represent “backbone” or essential elements of integrated care, enablers indicate activities and initiatives that help to facilitate and streamline effective integration, and outcomes represent what is hoped to be achieved through integrating care.

## Evaluation Findings

Our findings are presented within the 9 pillar framework and include observations within each domain about what went well, areas for improvement, and opportunities for the future of ETHP (See Table 1 for detailed findings and legend).

**Table 1:** Summary of findings.


9 PILLARS OF INTEGRATED CARE	WHAT WORKED WELL	QUOTES	OPPORTUNITIES FOR GROWTH	QUOTES

FUNDAMENTALS				

**Shared values and vision**	– leveraging trusted relationships – shared goals/common purpose – evolving from internal focus to collective focus	“When you sit at those tables, and try to solve problems together, that’s how you build your relationships. So it makes it stronger, and then when people have ideas, you’re able to kind of flow with it.”(CL) “Hospitals were inundated, and this fortress mentality wasn’t going to work…so with the whole ‘I need you and you need me, how can I help’ thinking opened a lot more doors.”(PO) “We did what was right, because it had to be done. Even sacrificing ourselves. Some people’s instincts would be protectionist. We helped as many people as we possibly could.” (CL) “I felt responsible and they (MGH team) made me feel that this would be okay, you will get through this” (PO) “(MGH IPAC team) came into to observe staff. They were coming to help, not take over. In other (LTC) homes, the reception of this team was not positive, but we recognized they were here to help.” (PO) “putting our hats aside and rowing in the same boat” (PO)	– expansion of newly created relationships – being more proactive	“We need to be proactive to start thinking about what our communities need”(PO) “…we have some disadvantaged populations, and we want to work together to figure out how to serve them best. This unifies us, brings us together, and is a big driver for creativity, innovation, and motivation to do something differently.”(CL) “Thinking about the health of a community through the partnership with schools and hospitals – we can be more proactive on population health.”(CHA)

**People as partners in care**	– incorporation of community feedback – building vaccine trust – leveraging social enterprise (homemade masks)	“The Community Health Ambassador role has worked very well from the beginning, because they not only reflected the diversity of the people in East Toronto, but also most of [them] were not new to the community, they were already active, involved in projects, committees – they were well known and trusted in the community.” (CHA) “I’m absolutely proud of the collaboration on the ground and addressing some of the unique needs of our community and providing them language and culturally sensitive information, and really giving the community the autonomy to guide us and decide how we should support them.”(PO) “Every Thursday we are doing mobile testing in every building, outreach to promote testing and vaccine clinics. People are really engaged with us.” (CHA)	– leveraging community partnerships for new and ongoing initiatives	“Not much space for partnering with community member patients and caregivers (starting to do it now), although observation and gathering feedback was part of the process.”(PO)

**System wide governance and leadership**	– system-wide leadership and governance – primary care leadership – pushing system boundaries – innovation, agility, and risk-taking	“We were able to adapt in real time to be out in front, finding options, getting testing to people.”(CL) “Would not have been able to do what I did without the full support of [hospital] leaders. [Name of Hospital CEO] said ‘I have your back’. This was huge.” (CL)	– increased focus on Social Determinants Of Health (SDOH) – create a governance model – recalibrate organizational relationships	“[We did not] fully catalyze our relationship with government agencies.”(PO) “A model of convening and collaboration is important, but we need a governance model that addresses true integration.”(CL) “[There are] challenges in working collaboratively with organizations that are at your level, or maybe a bit higher, and in the ranking of our healthcare system. I’m not saying that the hospital is higher than the community organization, but it might be perceived that all the resources goes to the hospital first, because we’re […] not putting much emphasis on the importance of community partners and community organizations”(PO)

**ENABLERS**				

**Workforce capacity and capability**	– sharing scarce resources – providing support to multiple sectors	“Can’t speak enough of [IPAC Champion], I could email, call every time I had a question.” (PO)	– reducing burnout by distributing work more effectively – development of a collaborative model for HHR planning and capacity	“We should’ve delegated. Same people doing everything was helpful, but exhausting.”(CL) “For collaboration to work …staff need to feel they are not losing control.”(CL) “We missed opportunities for reform… we could’ve done something about PSW wages.”(PO) “This was a field we had no knowledge of. How do we keep everyone safe?”(CL) “Better recognition of sacrifices made…e.g., say thank you, share success stories, share struggles in a safe place.”(PO)

**Digital solutions**	– data-driven response – virtual care and digital supports	“We saw everyone who came in, called everyone, we had a real pulse on what was happening, a sense of responding to peoples’ stories – challenges of self-isolating, etc really helped us plan.” (CL) “Data was shared by MGH and used for planning testing, clinics, vaccines, pop ups…[we used data] to plan response at micro, meso, macro levels.” (PO) “the City missed prioritizing Taylor Massey at the beginning, but we had data to show otherwise.” (CL) “Having virtual ward supports for home monitoring was helpful.” (CL)	– capacity for collection and analysis of population health data – leverage gains made in digital/virtual care during pandemic	“Digital component is key. There is funding. How can we really leverage this to be a foundation for the next decade?”(CL)

**Aligned payment systems**	– supply chain distribution (PPE) – shared capacity and resources	“They dropped the supplies off at our front door until we were able to manage our own PPE.” (PO) “We all believe an integrated system will drive better health outcomes and resources being used differently. The only way to be truly responsive is for providers to think about how they might operate differently. Not just with new funding, but with current funding.”(Gov)	– expand resources beyond anchor partners – new funding pitches for integrated care	“Individualized funding in a crisis does not work. And it speaks to that idea of how do you build integrated funding models, where you have fluidity and common goals and outcomes.”(PO)

**OUTCOMES**				

**Population health and local context**	– equity-focused response – addressed social determinants of health – new partnerships with community	“It was clear that we were prioritizing testing in areas where there were more barriers. [We] created structures to support local neighbourhood testing and ran multiple testing sites. This was very key.” (CL) “The vaccine testing strategy that changed everything was the pop up at Iqbal foods. We had no idea what would happen.” (PO) “We wrote letters to prompt the government on things like preparing for expanding testing, preparing to do 100,000 tests/day. When [the] Minister came, [we] gave her the truth about Armageddon coming, [but] there was no way to escalate. This was frustrating.” (CL)	– restructuring/alignment of healthcare and public health – increased focus on SDOH – sustainability	“You cannot achieve health outcomes without addressing social determinants. That’s the simplest. And I think the pandemic really highlighted this. If we didn’t have food security, people would not be able to self isolate. And the spread would have been much higher than what we have. The [way] we control this is by addressing social determinants of health, and you cannot accomplish health equity without addressing social determinants.”(CL)

**Resilient communities and new alliances**	– hyper-local focus and tailored solutions – capacity building – innovative community partnerships and community health ambassadors	“[We] knew early on this was a community issue not a hospital issue, so we needed to pull in our partners.” (CL) “The match between CHAs and community agencies was a match made in heaven. Prior to the pandemic, many of the agencies were working in silos. With the pandemic they joined hands together.” (CHA) “[We] respected and tapped into the knowledge residing within the [East Toronto] community.” (CL) “Shelter providers really appreciated our work and assistance. They felt that they were not abandoned. They felt we were accessible[…]. They appreciated no bureaucracy around our response. [ETHP] got a lot of shout out in the media.”(PO)	– creation of community outreach team – continue to build on new partnerships	“As members of an OHT, now that we develop a rapport with LTC partners, we should play a significant part in helping address some of the LTC challenges and how we deliver care for older adults, e.g., staff/resident ratio; standardized pay; physician care model; advanced care planning and palliative care; integration of acute, LTC and home care.”(PO)

**Transparency of progress, results and impacts**	– communication between partner organizations – sharing stories and media coverage	“[It] took us longer to react to the issues in LTC than we would have liked. We should’ve been working with that sector 4 weeks earlier than we did.” (CL) “[We] underestimated how hard it would be to work in Taylor-Massey. [We] took the vaccine rates very personally.” (PO)	– use of data-driven approaches – increased data flow – greater transparency	“There were factors beyond our control such as funding, but we could’ve allocated our existing resources in a better way such as investment in food security and mental health …as people really needed these services.”(PO)


Legend. Representative from a partner organization (PO) (i.e. schools, long-term care homes, community agencies, acute care) PO; community health ambassador (CHA); clinician leader (CL); provincial/municipal government (Gov) (includes public health).

### Fundamentals

#### Shared values and vision

Our data suggests that while the early stages of the pandemic saw organizations turning their efforts inward, existing and newly forged relationships were soon leveraged to combat a common enemy, COVID-19. Pre-existing relationships between members of different organizations were perceived as a key element as it enabled difficult conversations about resource sharing. New relationships with other sectors (e.g., schools, LTC, and public health) were also seen as a key factor in combating COVID-19. Early communication about policies and organizational approaches helped to create a more unified response across partners:

*“Hospitals were inundated, and this fortress mentality wasn’t going to work…so with the whole ‘I need you and you need me, how can I help’ thinking opened a lot more doors.”* (Representative from partner organization)

Looking ahead, respondents suggested that there is a need to harness the increased sense of shared vision, strong relationships, and values to continue building integrated health and social care, nurture existing and new partnerships, look for additional collaborative opportunities, and be proactive with the emerging needs of the community.

#### System-wide governance and leadership

Distributed, inclusive leadership across partners and people was perceived as core to the collective response. There was a mix of size and scale among partners who ‘kept punching above their weight,’ which translated into more capacity and support. New leaders were supported, and clinicians, primary care providers, and high-performing staff stood up or were tapped to lead the response. Early on, it became evident that the role of primary care was significant in leading the response. Adaptation and nimbleness in real time were other hallmarks of the response, which resulted in many provincial and Canadian ‘firsts’ for innovative approaches (e.g., take-home COVID-19 testing kits for schools).

*“We were able to adapt in real time to be out in front, finding options, getting testing to people.”* (Clinical leader)

Still, there were challenges, including working within the confines of provincial and municipal structures. Being on the ‘bleeding edge’ meant constant pushing and advocating for things to happen faster and tension with provincial governing authorities. This suggests an opportunity to recalibrate the relationship with municipal and provincial partners to create a shared vision for how to better collaborate in the future. Other opportunities for growth include an increasing collaborative focus on social determinants of health and scaling up efforts to include all neighbourhoods. Finally, interviewees identified an opportunity to create an integrated governance model to facilitate more advanced collaboration beyond COVID-19.

#### People as partners in health and healthcare

The community support for the collaborative response was perceived as strong, as demonstrated by the social enterprise of people sewing masks and many examples of local businesses donating food and gifts for front-line hospital staff – the community was engaged and wanted to contribute. This furthered the goal of providing equitable access. The most mentioned example by participants was the vaccine rollout co-designed with community members that focused on high needs populations, including seniors, Indigenous and racialized people, people with disabilities, and people with mental health and substance use challenges. Some of the vaccine clinics included local musicians to entertain people who were waiting in line.

*“Every Thursday we are doing mobile testing in every building, outreach to promote testing and vaccine clinics. People are really engaged with us.”* (Community Health Ambassador)

Regular forums for the community were an important way to build trust with schools, parents, and providers. Based on the success of the community engagement, participants believed that there was a missed opportunity for greater partnership with patients, caregivers, and community members in co-design earlier in the pandemic response.

### Enablers

#### Aligned payment systems

Partners observed an increased democratization, including weekly forums, open conversation and problem solving, as well as a willingness to realign resources. Participants listed a series of initiatives in which they shared resources. These included the centralized management of PPE and vaccine distribution, which was perceived as an enabler that resulted in efficient and trusted supply chains. Additionally, with support from partners, MGH expanded the bed footprint of the hospital by 25%. Partnerships were also created for integrated palliative support, home care, and expanded substance use support. In the future, opportunities to expand/realign resources and power beyond the six main anchor partners and new funding pitches could be explored. There is also room for incorporating learnings from integrated funding models implemented in Ontario [26] and other jurisdictions.

*“Individualized funding in a crisis does not work. And it speaks to that idea of how do you build integrated funding models, where you have fluidity and common goals and outcomes.”* (Representative from partner organization)

Progress toward aligned payment systems is heavily influenced by external factors (e.g., politics), but there might be a leadership role for ETHP in transforming healthcare delivery and payment.

#### Workforce capacity and capability

Scarce resources, including staff, were shared between organizations. This was enabled by communication among partners about best practice supports, shared health and human resources (HHR), and staff-related policies. Sharing knowledge and expertise was seen as a collaborative endeavor to build capacity across East Toronto. For example, participants mentioned that the Q&A sessions with the IPAC team and dedicated communications support facilitated the sharing of critical information between organizations. The local IPAC champion model was perceived as successful and impactful, and a critical way to extend IPAC resources and support across partners.

“Can’t speak enough of [IPAC Champion], I could email, call every time I had a question.” (Representative from partner organization)

While the level and connection to IPAC support for partners was well received, it was not intended as a long-term solution. Burnout has been an ongoing risk factor as the strategy for COVID-19 response relied on a small number of people working around the clock. Additionally, although the connection that was established between LTC and other settings is likely to have a long-term impact on how care is delivered (e.g., staffing ratios in LTC, nurse-led outreach teams, palliative care), some room for improvement remains (e.g., standardization of wages for personal support workers). Looking ahead, developing a collaborative model for HHR planning and capacity could address differential pay across partners and the ability to move staff between organizations. Further, building on what has already been done, maintaining a regional support model for IPAC, integrating clinical relationships with long-term care, and considering new roles (e.g., a medical director for community geriatrics) were perceived as key steps moving forward.

#### Digital solutions

Participants agreed that the East Toronto response leveraged local data to understand where to target testing and vaccination efforts. For instance, all COVID-19 tests and vaccinations were analyzed by postal code to proactively identify infection hotspots and areas with low vaccination rates.

*“The city missed prioritizing Taylor Massey at the beginning, but we had data to show otherwise.”* (Clinical Leader)

Additionally, virtual care options (e.g., virtual ward) were quickly implemented to safely deliver care services during outbreaks. Also, family physicians were connected to hospital systems and specialists using digital solutions to support patients in the community. Looking ahead, participants hope to see development of system-level capacity to collect and utilize population health data, as well as leveraging gains made in digital and virtual care as a foundation for the next decade. Actively looking for investment to transform our digital infrastructure was also seen as a priority.

### Outcomes

#### Resilient communities and new alliances

Prior to the pandemic, there was a pre-existing neighbourhood-based community outreach model to support high priority communities across Ontario (i.e. neighbourhoods with lower socio-economic status, higher population density, and a greater proportion of newcomers/immigrants) funded by the municipality and province. The high-priority communities strategy involves community organizations recruiting local residents to be ‘community health ambassadors’ who can provide connection, outreach, and education within their own communities. Using a ‘community-centred asset-based approach’ [27] ETHP assessed and leveraged the resources and connections already existing in our communities, including our community health ambassadors, to support our COVID outreach work.

*“The match between CHAs and community agencies was a match made in heaven. Prior to the pandemic, many of the agencies were working in silos. With the pandemic they joined hands together.”* (Community Health Ambassador)

Community ambassadors sharing messages, for instance, about masks, hand hygiene, and physical distancing early on was proven to be a more trusted source than healthcare workers. As a result, it was perceived that testing and vaccination efforts were extended to reach deep into communities and priority populations. The partnership also included co-design of outreach strategies tailored for priority neighbourhoods and populations to improve access. Innovative community partnerships were also leveraged for the rapid implementation of new models of care (e.g., COVID-19 community case management), which was seen as key to help address disruptions in regular services and support.

The key post-pandemic opportunity identified by participants was the creation of more formal community outreach capacity (e.g., cancer screening, working with schools to advance healthy habits and public, mental, and paediatric health).

#### Population health and local context

A main theme that emerged from the collective COVID-19 response was the importance of equity-based strategies to focus on improving access and ultimately population health. These focused on both high-risk populations (e.g., homebound seniors, people living in shelters) as well as priority neighbourhoods, and included activities like supporting self-isolation for low-income families by covering rent during the isolation period.

*“It was clear that we were prioritizing testing in areas where there were more barriers. [We] created structures to support local neighbourhood testing and ran multiple testing sites. This was very key.”* (Clinical Leader)

The COVID-19 response was seen as holistic in that it addressed broader social determinants of health (e.g., conducting testing and vaccinations at a food bank). Strategies were changed based on the community’s needs, particularly for increasing vaccine trust and improving access to vaccines. In addition, a significant amount of time and energy was spent advocating for the needs of residents and equitable access to care.

Going forward, an increased focus on social determinants of health is needed. A sustainable plan for infectious disease management and a focus on mental health and substance use were also identified as opportunities for future improvement.

#### Transparency of progress, results, and impacts

Dedicated communication resources and widespread sharing of information, including policies, were perceived to enable transparency and hence greater partnership. Participants noted that community partner resources were leveraged to facilitate communication in multiple languages in culturally appropriate ways. Despite these efforts, it was perceived that early communication could have been improved. For example, local news agencies ran stories about ETHP and their initiatives throughout the pandemic response; while the media coverage was overall positive, early media coverage on one of the harder hit neighborhoods was negative, not considering the socio-economic factors at play. Despite limited capacity to respond to all media requests, ETHP partners worked together to change the narrative and improve access to testing, vaccines, and other support for disadvantaged communities.

Going forward, a data driven approach is needed to measure progress toward ETHP’s goals, such as addressing ongoing health inequities for marginalized populations. Having an evaluation plan supported by a data governance strategy co-designed with communities was identified as a priority to showcase success and identify areas for improvement.

*“[We] underestimated how hard it would be to work in Taylor-Massey. [We] took the vaccine rates very personally.”* (Representative from partner organization)

Additionally, increased transparent reporting of impact and progress to partners and the public was seen as beneficial.

## Discussion

As in other jurisdictions, the COVID-19 pandemic was the catalyst for rapid change in how care is organized and delivered by the ETHP. The need for organizations to work together became acute, and the importance of prioritizing equity was undeniable as impacts of the pandemic compounded within already marginalized communities. In Toronto and across the globe, the social determinants of health played a large role in who acquired COVID-19, who could safely isolate, and who experienced the most adverse health effects after infection [28].

Integrated care was already a priority in East Toronto before the pandemic, but integrated care systems were still in their infancy. The integration of health and social care adds unique challenges (e.g., overcoming differences in legal frameworks, budgets, workplace cultures, accountability mechanisms, etc. [29]). Perhaps a testament to the scale of the challenge, a recent audit of health and social care integration within the UK’s National Health Service found that despite many years of advocacy and efforts, integration initiatives have yet to show clear evidence of producing higher quality care or cost-savings [30]. Nevertheless, the groundwork that had been laid at ETHP, including many informal relationships between members of different organizations, served as a jumping off point for integration during the pandemic. Even though organizational siloes still posed problems during the pandemic, most already saw the value of moving toward integration. Partnering with patients and community members was also a shared goal, but not all organizations could do it with ease. Partnerships between organizations with greater community connections and organizations that struggled with these connections were key for success, particularly in the vaccination effort where high levels of trust were needed. Community members themselves, including many volunteers living in priority areas, proved to be an invaluable asset to the pandemic response. The importance of developing relationships and trust with community members has also been emphasized by other Toronto-based integrated care researchers [31,32,33].

At the leadership and administrative level, achieving rapid, effective collaboration was essential to the ETHP response, particularly in the distribution of knowledge (e.g., about PPE), and resources including human health resources. The unique knowledge contained within each sector needed to be shared to inform the collective response. This challenged pre-existing organizational boundaries and brought to the fore differences in values, resources, and norms. The urgency of the pandemic response focused these collaborations on a common goal and allowed differences to be set aside and relationships to be built that may have otherwise been hindered by organizational differences. A need for aligned payment systems and better distribution of resources, particularly beyond ETHP anchor partners, was identified. These challenges are like those found in a recent scoping review of paramedics in integrated models of care [34], which emphasized the importance of a mobile and flexible workforce, cross-cutting service organizations (i.e., those that naturally interface with multiple sectors), the need for permissive regulation, and the role of funding models in whether value is assessed at a systems level.

### Post-pandemic Recovery and the “New Normal”

As the pandemic wanes, priorities are shifting from infection control and vaccination efforts back to the mainstay healthcare issues, now compounded by staff shortages, burnout, care delivery backlogs, and a host of other complexities. Without the urgency and focused attention that the pandemic demanded, more concerted efforts will be needed to maintain and grow relationships developed during the pandemic.

ETHP has started to build on what was learned during the pandemic and develop a roadmap for COVID-19 recovery. The province has identified quality improvement (QI) indicators for OHTs, including improving access to alternative levels of care for patients remaining in acute care beds after their acute care needs are met, reducing emergency department presentations of mental health and substance use, and increased cancer screening. Building on learnings from the COVID-19 response, three working groups were developed for each QI focus. Members include internal experts from across ETHP, including patients, caregivers, community ambassadors, clinicians, providers, researchers, and staff. Engagement sessions were held, and data helped participants to identify priority populations for each indicator. New alliances with external experts were developed to facilitate co-design of strategies to improve the QI indicators. Additionally, a learning health system steering committee is being created to plan and oversee the post COVID-19 recovery by implementing rapid cycles of evaluations and combining resources of evaluation, QI, digital, analytics, and research departments.

### International and Evidence Context

The East Toronto COVID-19 response shares many common themes with responses in other jurisdictions. Integrated care and learning health systems have been on the radar of healthcare organizations for some time, and the pandemic was a catalyst for change in many places. A learning health system in Cincinnati identified many similar barriers in their response to the pandemic [35]. This included reluctance to share data between organizations due to a competitive versus cooperative mentality, siloed nature of data and information exchange, misalignment between sectors and organizations, and poor data infrastructure. Issues related to equity are not unique to East Toronto as the COVID-19 pandemic exacerbated inequities all over the world, including those related to race/ethnicity, socioeconomic status, homelessness, disability, and ageing [36]. A survey of 208 staff in Sweden about their views on inter-organizational collaborations [37] found that insufficient knowledge about each other’s work settings was most commonly cited as a barrier to collaboration, and insufficient experience with collaboration was the second most cited barrier. Much of the ETHP pandemic response involved finding innovative ways to push past these same barriers, motivated by the urgency of the pandemic.

The East Toronto experience with integrated care is not likely to substantially differ from other settings where integrated care systems are emerging and therefore these findings should be relevant and useful in jurisdictions across the globe. Given the wide range of organizations and sectors that participated in the ETHP pandemic response, these learnings are also scalable and could be applied in narrower (e.g., disease-specific) or broader (e.g., state or national level) applications of integrated care.

### Strengths and Limitations

This analysis was strengthened by the participation of individuals representing a wide range of organizations across the continuum of care. The study is also strong in its broad applicability and transferability to other settings, particularly as it relates to issues around equity and challenges associated with transforming siloed health systems into integrated systems. Additionally, conceptualizing the nine pillars of integrated care within the categories of fundamentals, enablers, and outcomes added value to this analysis and may be useful for other researchers.

There are also some limitations. While there was a diversity of voices represented in the interviews, more diversity could have been achieved (e.g., interviewing more community members as well as patients who experienced COVID-19). With more resources and time, more interviews or focus groups could have been conducted. Researchers and respondents were key actors in the pandemic response, so time restrictions were especially pronounced during this project. A secondary analysis of data could be beneficial to understand differences and/or similarities across sectors and professions. Quantitative methods (e.g., surveys) may have also helped to gather information from a larger proportion of individuals involved in ETHP’s pandemic response.

**Lessons Learned**

- Local leaders with exceptional vision and communication skills are essential
- Equity cannot be achieved without direct collaboration with community members
- Lack of consistency in pay, labour restrictions, and practises between organizations is a barrier to an integrated response
- Informal relationships between members of different organizations were the backbone of initial collaboration efforts
- A shared vision is critical – in this case, a common enemy (COVID-19) unified organizations and accelerated pre-existing collaboration efforts

## Conclusion

This study described and analyzed the ETHP COVID-19 response and tied learnings to the IFIC’s nine pillars of integrated care. The experience of ETHP, an emerging integrated care system, may serve as a useful guide for other integrated care systems that are working through the initial stages of integration. Many of the themes, including those around relationship building, equity, and organizational structures, are broadly applicable. Moving forward, more research related to implementation challenges and solutions would be welcome. Achieving effective integrated care is an immense practical challenge and many could benefit from practical tools and guidance. For similar reasons, more research related to the evaluation of integrated care systems would be of value.

## Additional File

The additional file for this article can be found as follows:

10.5334/ijic.7014.s1 Appendix A. Interview guide.
