# Supplementary material for: Evaluating an Integrated Local System Response to the COVID-19 Pandemic: Case Study of East Toronto Health Partners
Source: Int J Integr Care. 2023 Jun 22;23(2):31. doi: 10.5334/ijic.7014 (PMC10289038; doi:10.5334/ijic.7014)
Supplement: Appendix A. — Interview guide. [file ijic-23-2-7014-s1.pdf]

Appendix A- Interview guide

Section 1: Introduction

The objective of this research is to understand and document the impact of the East Toronto Health Partners (ETHP) integrated response to the COVID-19 pandemic. This research is being conducted by researchers affiliated with ETHP and the University of Toronto.

This research uses a mixed-methods approach that includes a quantitative analysis of data related to the COVID-19 response in East Toronto, including but not limited to, COVID-19 testing and vaccination rates by populations, hospitalization data, staffing numbers, and in-kind contributions. In addition, researchers will also be interviewing up to 30 key informants who have been identified as playing a critical role in the East Toronto COVID-19 response. The interviews are designed to learn about the ETHP COVID-19 strategy and impact of the response, including how the ETHP strategy was perceived by partners and community members. Lessons learned from this evaluation will be used to inform the ongoing COVID-19 strategy as well as next steps in the development of integrated health and social care (Ontario Health Team) in East Toronto.

Question 1: Please share your reflections on the East Toronto response starting with the earliest days of the pandemic as well as how the response evolved over time.

Prompts

- What was your role/your organization’s role in the pandemic?
- Thinking back to the earliest days in Wave 1 of the pandemic, how would you describe the initial East Toronto response?
- How did the East Toronto response change/evolve over time?

Question 2: What are the critical elements/building blocks that have defined the East Toronto response?

Prompts

- Who led the response? How have decisions been made?
- What are mechanisms/processes/structures that we used to coordinate our response?
- Which mechanisms/processes/structures had the most significant impact from your perspective – for you, your organization, patients/community?
- How were partners/community members engaged?
- What kind of data were collected? How was the data used?

**Question 3: What are you most proud of? Where could ETHP have done better?**<br><br>Prompts<br><br>• What aspects of the ETHP response worked well?<br>• What are you most proud of?<br>• Where did ETHP miss the mark? (i.e. make mistakes or did not respond sufficiently)<br>• What do you wish had been done differently in hindsight?
**Question 4: How would you describe the collaboration among partners and with the community? How was the East Toronto response perceived?**<br><br>Prompts<br><br>• How was the East Toronto response perceived by our community? By our partners?<br>• Describe the interaction with/support from regional partners (Ontario Health/Toronto Region, Toronto Public Health, City of Toronto, others)
**Question 5: What are the implications for the future/next steps in the development of integrated health and social care (OHT) in East Toronto?**<br><br>Prompts<br><br>● Where does East Toronto go from here?<br>● What are the most critical lessons learned that you would like to share?<br>● What aspects of the East Toronto response should be sustained or accelerated?
**Question 6: Conclusions and Wrap-Up**<br><br>Is there anything that we did not discuss that you think is relevant/important for evaluating the East Toronto COVID-19 response?
